# Oxidative/Nitrosative Stress and Brain Involvement in Sepsis: A Relationship Supported by Immunohistochemistry

**DOI:** 10.3390/medicina60121949

**Published:** 2024-11-26

**Authors:** Giuseppe Bertozzi, Michela Ferrara, Mariagrazia Calvano, Natascha Pascale, Aldo Di Fazio

**Affiliations:** 1 SIC Medicina Legale, Via Potito Petrone, 85100 Potenza, Italy; michelaferrara13@gmail.com (M.F.); mg.calvano@gmail.com (M.C.); nataschapascale@gmail.com (N.P.); aldodifaziomedicolegale@gmail.com (A.D.F.); 2 Department of Anatomical, Histological, Forensic and Orthopaedic Sciences, Sapienza University of Rome, Viale Regina Elena 336, 00185 Rome, Italy

**Keywords:** 8-OHdG, NOX-2, NT, iNOS, brain, nitrosative stress, immunohistochemistry, sepsis

## Abstract

*Background and Objectives*: A large amount of recent evidence suggests that cellular inability to consume oxygen could play a notable part in promoting sepsis as a consequence of mitochondrial dysfunction and oxidative stress. The latter could, in fact, represent a fundamental stage in the evolution of the “natural history” of sepsis. Following a study previously conducted by the same working group on heart samples, the present research project aims to evaluate, through an immunohistochemical study, the existence and/or extent of oxidative stress in the brains of subjects who died due to sepsis and define, after reviewing the literature, its contribution to the septic process to support the use of medications aimed at correcting redox anomalies in the management of septic patients. *Materials and Methods*: 10 cases of subjects who died in healthcare facilities with ante-mortem clinical-laboratory signs that allowed the diagnosis of septic shock were selected as case studies, and 1 case of a subject who died immediately following a road traffic accident was used as a negative control. Samples of the cerebral cortex were then taken, fixed in formalin, and subjected to sections on which an immunohistochemical study was performed using anti-NOX-2, NT, iNOS, and 8-OHdG antibodies. *Results*: The results emerging from the present study demonstrate that despite a variable expressivity for the NT, iNOS, and NOX2 markers, the brain samples demonstrated univocal and high positivity for the 8-OHdG marker. *Conclusions*: This would allow us to hypothesize how, regardless of the mechanism of production of ROS and NOS (iNOS or NOX2 mediated) and the pathophysiological mechanisms that are triggered during sepsis, oxidative damage to DNA represents the event to which this whole process leads and, in fact, in the literature, is directly correlated to sepsis-dependent mortality. Neurons, conversely, appear to be more sensitive to oxidative stress because of a low number of protective or scavenger molecules (catalase, glutathione peroxidase, GSH, or vitamin E). Therefore, despite reduced production, the manifestation of the damage remains high. This evidence, together with that of the previous study, can only support the introduction of substances with an antioxidant function in the guidelines for the treatment of sepsis.

## 1. Introduction

Sepsis is a global health problem causing a life-threatening, multiorgan, progressive failure triggered by a dysregulated host response to infection [1]. There are approximately 47–50 million cases of sepsis worldwide each year, 80% of which occur in the community [2]. Forty percent of cases occur in children under the age of 5. In Europe, approximately 700,000 cases of sepsis are reported. Globally, 1 in 5 deaths are associated with sepsis, resulting in at least 11 million deaths each year. Additionally, scientific literature shows that septic patients developed in up to 50% of survivors persistent physical, cognitive, and psychological sequelae such that they have assumed the nosological classification of post-septic syndrome. In Italy, the number of death certificates that reported sepsis as the main cause of *exitus* augmented from 18,939 in 2003 to 49,010 in 2015, that is to say, respectively, from 3 to 8% of all deaths recorded. Consequently, sepsis claims the attention not only of clinical experts but also of public health stakeholders, so much so that every year, all over the world, on September 13th, World Sepsis Day is celebrated, a campaign promoted by the World Health Organization (WHO) [3]. These attempts to raise awareness among the population about those subgroups that present particular risks, such as people with chronic lung, liver, and heart diseases, with splenectomy or immunodeficiency, newborns and infants, and over-60-year-old adults [4]. Individuals with cancer have a 10 times greater risk of sepsis and a 43% greater chance of septic death compared to non-cancer patients, as well as smoking increases the risk of respiratory infection and, consequently, sepsis.

Although sepsis is a dysregulated multiorgan response to infection, causing organ impairment, and despite the progress in our knowledge of its “natural history” and the best therapeutic modalities to date, the full understanding of its physiopathology is not there, and consequently, the diagnostic-therapeutic translation also requires further investigation [5,6]. Among the many processes that contribute to sepsis, such as inflammation, hypoxia, and organ disorders, have been linked to oxidative/nitrosative stress [7]. However, following previous work by the same research group in which the cardiac pattern was investigated, and which demonstrated the oxidative stress-related insult in cardiomyocytes [8], the present study aims to evaluate through an immunohistochemical analysis the involvement of reactive oxygen species in the pathophysiological processes, particularly affecting the brain.

## 2. Materials and Methods

### 2.1. Case Selection

10 cases, died in healthcare facilities with antemortem medical and laboratory data that allowed the diagnosis of septic shock, were selected. A subject was also included as a negative control (NC): a 30-year-old woman who died immediately following a road traffic accident with null family and personal cardiac and neurological history and no cardio-cerebrovascular risk factors.

All cases identified by this study come from judicial autopsies. As regards the cases of sepsis, they were all placed in a cold storage room, following the mortuary police regulation to prevent the progression of putrefactive processes, and the autopsy was performed within a week of death. The control case underwent an autopsy 24 h after death without staying in a cold chamber. The selection result is summarized in Table 1.

### 2.2. Immunohistochemical Analysis

Random brain samples were taken specifically from the cerebral cortex. All nervous tissue samples were subjected to treatment with a formalin solution (at 10%) and embedded in paraffin. The sections, approximately 4 microns thick, were made, and the immunohistochemical study was conducted using the following antibodies: gp91-phox (NOX-2), nitrotyrosine (NT), nitric oxide synthases-2 (iNOS), and 8-hidroxy-2′-deoxyguanosine (8-OHdG).

The rationale for the selection of the above antibodies was as follows:

- The term NOX refers specifically to the catalytic transmembrane protein (gp91-phox) and, by extension, to the entire multiprotein enzyme complex (composed of two protein subunits: p22-phox and p91-phox). NOX2, in detail, is the phagocytic NADPH oxidase, the most potent source of endogenous O^2−^ production [9];
- Nitrotyrosine (NT) results from the nitration of protein-bound tyrosine residues by reactive peroxynitrite molecules through the substitution of a hydrogen molecule with a nitro group in the phenolic ring of tyrosine residues. Nitration of proteins at the subcellular level causes conformational changes that damage the cytoskeleton and cause degeneration [10];
- Inducible nitric oxide synthase (iNOS) has been identified as the primary intermediary molecule in the production process of NO, which, together with its reactive species, can switch the redox cell balance through oxidative reactions, causing tissue damage [11];
- 8-Hydroxydeoxyguanosine (8OHdG), a typical compound of oxidative DNA damage, is made by direct oxidation of the guanine base, resulting in the addition of a hydroxyl radical to C-8 of the imidazole ring [12]. Compared to adenine and pyrimidines, this nucleobase is more susceptible to oxidative modifications in both RNA and DNA because of its low redox potential.

Immunohistochemical examination was based on a semi-quantitative screening, according to the ordinal method [13,14,15], on six random microscopic fields with original magnification 40× for each tissue section, conducted by two operators, according to a 0–4 scale, graphically translated as follows: −: negative immunoreactivity (0%); +: slight immunopositivity in spotted cells (10%); ++: immunopositivity up to one-third of the cells (33%); +++: immunopositivity up to two-thirds of the cells (70%); and ++++: strong immunopositivity in most or all cells (100%). In the case of divergent scores, a third observer decided the final category.

## 3. Results

**Figure 1:**
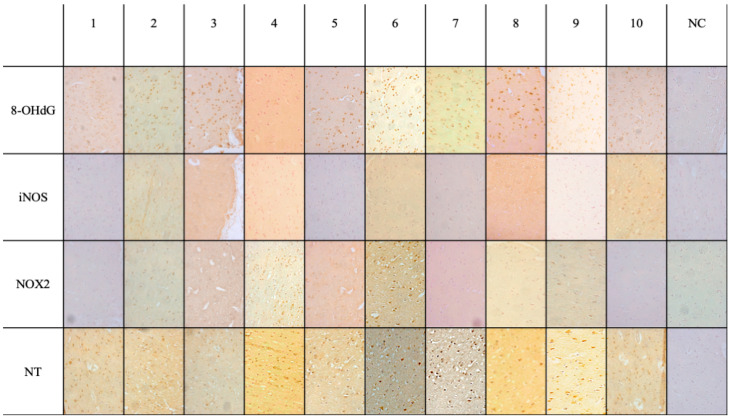
Immunohistochemical results for the sepsis group (20×) showing intense immunopositivity with 8-OHdG-antibody; substantial immunopositivity with NT; NOX-2 and iNOS mild to moderate immunopositivity.

Microscopic analysis of brain samples showed positivity, as in Figure 1, the results of which are summarized in the following synoptic table (Table 2).

Intense immunopositivity (Figure 1) was detected as far as 8-OHdG is concerned, quantified as more than 70% of cells in the septic group (A–C). In addition, intense and substantial immunopositivity was demonstrated with NT in the group with sepsis. NOX-2 and iNOS showed mild to moderate immunopositivity.

## 4. Discussion

The present work was born with the aim of investigating, through immunohistochemical study, the possible increase in oxidative/nitrosative stress (OS/NS) through its products and enzymatic upregulation in brain samples taken from subjects whose cause of death was defined ante-mortem and with certainty as sepsis. The clear and significant result is, in fact, represented by the intense positivity in the samples treated with markers to evaluate the expression of i-NOS, NOX2, and NT, which for all three antigens was significantly increased in the subjects of the sepsis group compared to the case check. This reaction is also associated with increased 8-OHdG, thus supporting the study’s hypothesis that OS/NS is a key factor in brain involvement in sepsis-related death [16,17,18]. The “upstream” difference in the immunopositivity of the three previously described markers could reside in the different expression of the oxidative pattern between viral and bacterial infection as well as in the different entity of the mechanisms that determine them (for example, endotoxinemia and TNFα are strictly related to nitrosative stress) [19,20,21].

Moreover, in the present study, it seems useful to report how the results have shown that in the face of a variable expressivity for the NT, iNOS, and NOX2 markers, the brain samples have shown unequivocal and high positivity for the 8-OHdG marker. This would suggest that independently of the mechanism of ROS and NOS production (iNOS- or NOX2-mediated) and the physiopathological mechanisms that are triggered during sepsis and that will be discussed in detail by organ in the following paragraphs, oxidative damage to DNA represents the event to which this entire process leads and, in fact, in literature, is directly related to sepsis-dependent mortality [22,23].

Furthermore, neurons appear to be particularly sensitive to oxidative stress; they present a low number of protective molecules or scavengers for free radicals (catalase, glutathione peroxidase, GSH, or vitamin E) [24]. Furthermore, brain parenchyma contains: (i) noteworthy quantities of unsaturated fatty acids and polyunsaturated fatty acids, which are particularly susceptible to free radical effects or lipid peroxidation, and (ii) high amounts of iron, which may be directly involved in free radical damage [25]. Therefore, in the face of reduced production (levels in the present study from mild to moderate of iNOS and NOX2), the manifestation of the damage remains high-elevated (NT and 8OHdG).

**Figure 2:**
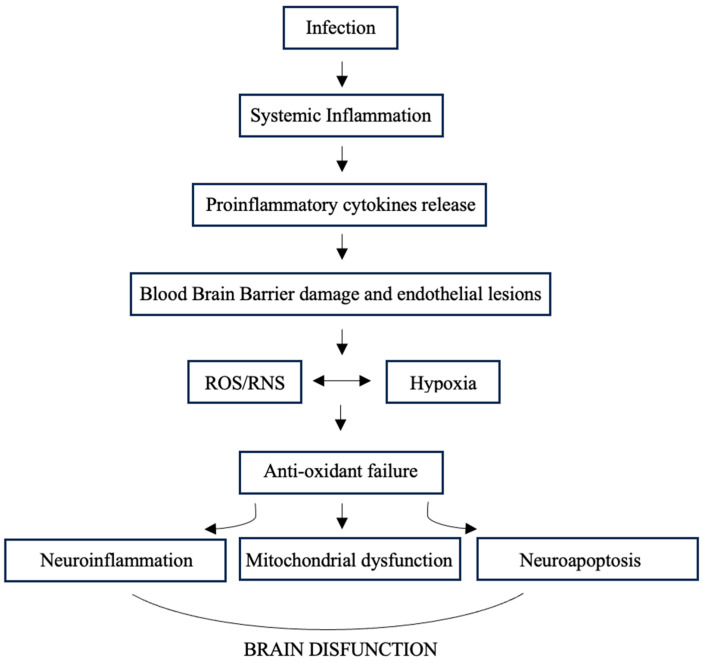
Model of brain dysfunction during sepsis. Systemic inflammation following infection leads to the release of proinflammatory cytokines that cause damage to the blood-brain barrier and widespread endothelial damage. These in turn lead to the generation of reactive oxygen and nitrogen species, which trigger lipid peroxidation chain reactions, exacerbated by the limited antioxidant capacity of the brain. The result is the induction of neuroinflammation and the inhibition of the mitochondrial electron transport chain, which increases mitochondrial release of free radicals, leading to mitochondrial dysfunction, leaving neurons in a state of “cytopathic hypoxia”. All these alterations, together with predominantly Ca^2+^-mediated apoptosis, cause brain impairment.

In detail, the review of the literature supports the results of the present study. The central nervous system represents a site that can be a source as well as a target victim of sepsis through a propagated immunoinflammatory dyshomeostasis and altered dynamics of cerebral blood flow (Figure 2) [26,27].

A major consequence of systemic inflammatory response syndrome in the case of sepsis involves the increased production of neuroinflammatory cytokines, which can lead to damage to the blood-brain barrier (BBB), alterations in neurotransmission, and instability in the functioning of neurohormones, such as acetylcholine, γ-aminobutyric acid, β-endorphin, and corticotropin-releasing hormone. In sepsis, structural damage to the blood-brain barrier, cerebrovascular endothelial membrane, and surrounding neurons is accompanied by a local response that is translated into an increase in intrathecal cytokine concentrations, enhancing the whole process: increased expression of tumor necrosis factor (TNF)-α, primarily at the periventricular level, and secondly involving glial components with induction of glial expression of interleukin (IL)-1β and IL-6 [28]. However, it is not possible to dissociate inflammation from oxidative-nitrosative stress. The known biological half-life of ROS/RNS is relatively short compared to that of cytokines, suggesting that free radicals function primarily as inducers, rather than simple propagators of inflammation. This is also in light of the fact that radical-mediated disruption of the blood-brain barrier allows access to the brain parenchyma to the products of oxidative metabolism, which then induces an initially focal inflammation during sepsis and subsequently exacerbates systemic inflammation [29].

All this culminates in a pathological condition known as sepsis-associated encephalopathy (SAE), characterized by vascular and endothelial damage, disruption of brain signaling, and death and ultimate degeneration of neuronal cells by apoptosis. In detail, during SAE, to balance the reduction in functional capillary density and consequent hypoxia, the organism tries to activate heterogeneous microvascular perfusion, which is insufficient. Furthermore, a disproportion between reactive oxygen species (ROS) and reactive nitrogen species (RNS) [7], induced by sepsis, stimulates a vicious circle of lipid peroxidation reactions through modification of the antioxidant cycle of neuronal protection and blood vessels. More importantly, oxidative imbalance causes mitochondrial dysfunction through an altered mitochondrial membrane potential and nitration of mitochondrial proteins, an inefficient activity of the electron transport chain. In detail, in fact, ROS and decreased glutathione levels linked to atypical mitochondrial activity noticeably affect the ATP synthase complex and, consequently, ATP production. Furthermore, competent oxidative metabolism depends on the capacity of electron transport chain function and oxidative phosphorylation through a proton gradient that occurs via the inner mitochondrial membrane. Oxygen radicals in the form of O^2−^ and lipid peroxides activate uncoupling proteins, which increase the permeability of protons across the inner mitochondrial membrane, resulting in protons entering the mitochondrial matrix to combine with electrons to form water in an exothermic reaction generating ATP from ADP. This uncoupling of oxidative phosphorylation in brain mitochondria causes a condition known as “cytopathic hypoxia”, meaning that mitochondrial incapacity to utilize oxygen is independent and respectful of its bioavailability. Additionally, ONOO-radical can introduce disruptions into DNA, thereby triggering the activation of various repair processes, including the NAD+-consuming enzyme nuclear polymerase, which increases cellular metabolic demands. This can result in neuronal bioenergetic breakdown. This puts a strain on cellular energy metabolism.

The site mostly involved in this process is in the centers of neurogenesis, mainly in the hippocampus and in the cerebral cortex [30] (the latter on which this study was conducted), where cells carrying all these alterations undergo death by apoptosis. Free radicals, in fact, can induce mitochondria-dependent apoptosis, compromising the integrity and function of the membrane in mitochondria and in the endoplasmic reticulum with an increase in intracellular Ca^2+^, which induces permeability of the transition pores in neuronal mitochondria, translocation of cytochrome c from the internal mitochondrial membrane to the cytoplasm inducing the apoptotic cascade [31]. Furthermore, the increase in ROS causes an increase in iron content and alterations in the proteins GPX4, ACSL4, and SLC7A11, recognizing the role of ferroptosis in sepsis-related brain degeneration [9]. Ferroptosis is a specific process of programmed cell death, recently the object of particular attention due to its unique morphological, genetic, and biochemical characteristics, and so called because it involves the dysfunctional metabolism of intracellular iron, which causes lipid peroxidation [32]. In detail, ferrous iron, which is produced as a result of the strengthening of the oxidative pathway already described and, therefore, whose increase is exponential during sepsis, interacts with polyunsaturated fatty acids in the cell membranes, providing free electrons through the reaction of Fenton, producing lipid hydroperoxides, resulting in excessive intracellular accumulation of these waste products, which triggers cellular ferroptosis. In physiological conditions, the correct redox state of the cells and the organism is such that the oxidative system consisting of iron ions, the Fenton reaction, and ROS and the antioxidant system consisting of glutathione peroxidase 4 (GPX4), glutathione, and the Xc system are neutralized between them [33]. It should be noted that the Xc-system has the function of exchanging cystine and glutamate from inside to outside the cell, with cystine then reduced to cysteine, which is involved in the biosynthesis of reduced glutathione, a well-known antioxidant molecule, and it is easy to imagine the consequences. During sepsis, ferroptosis is exacerbated because, on the one hand, it promotes infection by providing iron as a raw material for bacterial multiplication and, on the other hand, inhibits the immune response [34]. Indeed, cells such as macrophages, T cells, and B cells suffer from ferroptosis themselves, resulting in decreased number and function, and the dead ferroptosis cells, in turn, can be identified by immune cells and then trigger a series of inflammatory or specific immune responses. Moreover, exosome-derived lncRNA NEAT1 exacerbates SAE-related sepsis by promoting ferroptosis. In detail, in the study by Wei et al. [35], the miR-9-5p/transferrin receptor (TFRC) and glutamic-oxaloacetic transaminase 1 (GOT1) axes appear to be involved. On the other hand, Ferrostatin-1 (Fer-1) treatment promotes the expression of tight junction proteins, implying that inhibition of ferroptosis may act by inhibiting BBB disruption caused by sepsis, acting on a transcription factor called Nrf2, which belongs to a signaling pathway called Nrf-ARE, composed, in addition, of Kelch-like ECH-associated protein 1 (Keap1) and the antioxidant response element (ARE) [36]. When Nrf2 is linked to Keap1, it is usually inactive, and the actin-related inhibitory protein Keap1 traps Nrf2 in the cytosol in normal conditions. However, the downstream genes HO-1, CAT-1, and NQO-1 of Nrf2, a master regulator of the antioxidant response, can prevent lipid peroxidation and ferroptosis. Furthermore, Guo et al. found that melatonin therapy, with antioxidant functions, improved neuronal ferroptosis and promoted hippocampal neuronal survival via the Nrf2/GPX4 axis, confirming the key role of NRF2 in regulating ferroptotic death [37]. Furthermore, Fer-1 treatment blocks glutamate excitotoxicity by blocking the Xc-system and glutamate receptor N-methyl-D-aspartate receptor subunit 2, and these processes ultimately protect synaptic and neuronal integrity during SAE and improve SAE outcomes [38]. Furthermore, a role in sepsis-induced brain neurodegeneration would also be related to a concomitant sepsis-mediated activation of the P38 signal, increasing MAPKAPK2 phosphorylation and downstreaming P38 [39].

The detailed attention to ferroptosis, which is far from a mere in-depth dissertation that could be excessive compared to other types of cellular damage induced by oxidative stress, takes on importance for the possible and eventual therapeutic choices. Specifically on ascorbic acid, a mechanism that has been used for the destruction of tumor cells [40]. Ascorbic acid (AA) is the redox form of vitamin C and is a water-soluble antioxidant, a cofactor of multiple enzymatic reactions, not produced endogenously in the body, and exogenous food sources are needed for the necessary intake [41,42]. AA absorption occurs through the activity of the SVCT1 Na^+^-AA transporter. Low levels have been found both in patients with multiorgan failure and with increased lipid peroxides. Results from animal models have shown that AA improves edema and hypotension, as well as arteriolar reactivity and capillary blood flow [43]. In a phase I safety study of intravenous AA in patients with severe sepsis, the infusion was found to be safe and well tolerated [44]. In a retrospective analysis of the combination of hydrocortisone, vitamin C, and thiamine for the treatment of severe sepsis and septic shock, hospital mortality was 8.5% in the treatment group versus 40.4% in the control, and none of the study subjects developed progression of organ failure [45]. In addition, it can minimize damage to the blood-brain barrier by defending the endothelial barrier and preserving capillaries. However, AA induces cellular morphological changes in mitochondria associated with ferroptosis [46]. AA-induced ferroptosis is accompanied by glutathione depletion and increased levels of ferrous ions Fe^2+^, reactive oxygen species, and malondialdehyde, which are in turn potentiated by deferoxamine and N-acetylcysteine (NAC). The latter is a well-known drug with antioxidant activity capable not only of enhancing the action of GSH but also of promoting the availability of cysteine and, therefore, the synthesis of GSH itself [47]. Studies on humans have shown that the administration of NAC can significantly increase blood flow and can increase the phagocytic function of neutrophils in cases of sepsis or SIRS. The result is a reduction in the relative expression of signal transducer and activator of transcription 3 (STAT3) and glutathione peroxidase 4 mRNA with effects to be evaluated in the clinical setting in the management of patients with sepsis.

SAE also affects neurotransmission, mainly through altered profiles of norepinephrine, 3,4-dihydroxyphenylacetic acid, homovanillic acid, and catecholamines; increased metabolism of serotonin and its final metabolite, 5-hydroxyindoleacetic acid (5HIAA); and an increased serotonin/5HIAA ratio [48]. Finally, a glutamate-mediated input of vagal afferents also regulates autonomic functions with induced excitotoxicity [7]. All this is associated with the deprivation of the brain in its oxygen demand due to compromised microvascular regulation and cerebral hypoxia, culminating in trauma and permanent ischemic stroke. Thus, SAE involves oxidative, bioenergetic, and mitochondrial dysfunctions in the brain, suggesting that SAE is based on a cross-talk between autophagy, neuroinflammation, and stress.

In addition to the effects already described for ascorbic acid and NAC, several strategies have been used to reduce oxidative stress generated in mitochondria. The ability of lipophilic cations to accumulate in mitochondria makes them good candidates. MitoQ (ubiquinone attached to a triphenylphosphonium cation) protects cells from peroxide-induced apoptosis, reduces oxidative stress, and protects mitochondria from damage, resulting in lower rates of ROS formation and maintenance of mitochondrial membrane potential [44,45]. The hypothesis that MitoQ administration could prevent endotoxin-induced reductions in cardiac mitochondrial and contractile function was tested in adult rodents.

The use of superoxide dismutase (SOD) mimetics has also been shown to be useful: (1) M40401 is able to improve vascular responsiveness to vasopressors and reduce cytokine production; (2) MnIIITE-2-PyP5+ enters mitochondria and exerts its antioxidant action there [49].

The crucial role of NO in the development of sepsis and organ dysfunction has led to the implementation of therapeutic strategies capable of reducing NO levels through iNOS inhibition [50]. Treatment with the selective iNOS inhibitor, aminoguanidine, inhibited sepsis-induced plasma nitrate/nitrite concentrations, reduced LPS-induced bacterial translocation by improving intestinal hyperpermeability, improved oxygen consumption rate, and prevented hypotension without affecting cardiac output. An interesting alternative is ketanserin, a serotonin receptor antagonist, which causes an inhibition of iNOS expression through the MEK/ERK pathway with consequent improvement of microcirculatory perfusion.

Among the possible substances whose use should be evaluated in cases of sepsis, the aforementioned melatonin or N-acetyl-5-methoxy-tryptamine could also be considered, synthesized from the amino acid tryptophan in the pineal gland and secreted into the cerebrospinal fluid [51,52,53]. It has demonstrated anti-inflammatory, anti-apoptotic, and antioxidant effects for oxygen and nitrogen radical species and protection of mitochondrial dysfunction. In detail, melatonin can: restore mitochondrial ATP production; reduce markers of inflammation and oxidative stress; influence the determination of lower concentrations of lipid peroxidation products. It also has antioxidant properties directed against ROS (including OH-, H_2_O_2_, singlet oxygen, and peroxynitrite-HNOO-) and promotes the activation of various antioxidants by inhibiting the pro-oxidant activity of some enzymes.

Antiapoptotic, antiferroptotic, and immunomodulatory properties are also achieved by blocking the NF-κB-induced pathway, with protection of cells from hypoxia and hemoglobin [54,55]. Melatonin has also been observed to reduce D-dimer levels in sepsis- and COVID-19-induced coagulopathy, failing to prevent DIC in sepsis [56].

Resveratrol C_14_H_12_O_3_ is a polyphenol produced by plants in response to exogenous stimuli, such as ultraviolet light, mechanical damage, or fungal infections [57]. Its name comes from the roots of white hellebore, where it was first identified in 1940. It is able to inhibit NF-kB activation and downregulate nitric oxide synthase, adhesion molecules, and tumor necrosis factor-α [58,59]. The improvement of endogenous antioxidant capacity, to which exogenous resveratrol contributes, promotes an improvement of sepsis-related organ damage, reducing that related to oxidative stress: inhibiting erythrocyte lipid peroxidation and catalase activity, reducing nitric oxide release, downregulating malondialdehyde levels, maintaining iron homeostasis, increasing phagocytosis, regulating the inflammatory state, and inhibiting the development of endotoxin tolerance.

Also, α-tocopherol, or vitamin E, is a fat-soluble compound, omnipresent in vegetable oils, with potent antioxidant properties, acting by breaking the lipid peroxide chain at the membrane-water interface, decreasing brain levels of MDA produced by lipids induced by oxidative/nitrosative peroxidation stress, and reducing the concentration of proinflammatory cytokines TNF-α and IL-6 [60,61,62].

It is worth mentioning here that, unlike sepsis, the post-injury neuroinflammatory response elicited during traumatic brain injury (TBI) also involves pro-inflammatory cytokines and free radical formation; however, the pathological changes induced by TBI, and for this reason, specifically include diffuse axonal injury (DAI), damage to the neurovascular compartment, and disruption of the neuronal network, resulting in cellular apoptosis or necrosis [63]. In fact, TBI, on the other hand, has also been linked to oxidative stress damage as a secondary phenomenon, so much so that quantifying biomarkers of oxidative stress and antioxidant status of serum of subjects who have experienced it has been identified as a possible marker of trauma severity and prognosis [64,65]. However, and precisely, thanks to the complex of manifestations that distinguish the two phenomena, in theory the post-mortem diagnosis, useful for the forensic identification of the cause of death in those cases in which it is not possible to access the clinical documentation of the patients, could, through immunohistochemical or biochemical tests, focus on the identification of specific markers of head trauma, compared to sepsis, such as aquaporins, S100 calcium-binding protein β (S100β), and neuron-specific enolase (NSE) [66,67]. That is, although it is a moment of common arrival, further studies are necessary in order to compare the extent of oxidative stress in the two mechanisms (sepsis vs. TBI) and any markers more typical of one compared to the other.

### Limitations

A weakness of the study is that it only had one case as a control sample. This prevents us from analyzing the effects that different post-mortem time intervals can generate in the immunohistochemical reaction of the selected markers. This is because as the PMI increases, the degradation of the protein molecules appears, which alters the antigens and determines their migration to a site other than the original one. Furthermore, a false positivity may occur due to the greater binding of the antibodies to altered epitopes [68]. This condition may also be affected by freezing related to storage in a cold room.

Another limitation for the purpose of generalizing the results obtained in this study is that the sepsis group consists of only 10 samples, which makes further studies with larger samples necessary to give greater reliability to the statistical investigation.

## 5. Conclusions

The present study focused on the possibility of demonstrating the involvement of the brain as a target of organ damage induced by sepsis. To this end, the expression of typical markers of oxidative stress was studied through an immunohistochemical study. To date, to the best of our knowledge, it would represent the only study in the literature demonstrating such a relationship in subjects in which the cause of death was attributed with certainty to sepsis following autopsy findings. This evidence, together with the data provided by the previous study, supports the need to introduce substances with antioxidant function in the routine treatment of sepsis. However, the choice of drugs should also be carefully evaluated by balancing the risk of exacerbating sepsis/benefit in counteracting it; therefore, further specific studies are necessary.

## Figures and Tables

**Table 1:** Case selection.

Case	Sex	Age	Origin of Septic Shock
1	F	57	Pneumonia
2	M	62	Fournier’s gangrene
3	M	68	Peritonitis due to bowel perforation
4	F	75	Pneumonia due to VAP
5	M	27	Pneumonia
6	M	45	COVID-19 pneumonia
7	M	68	Cholecystitis
8	F	85	Pneumonia
9	M	62	Peritonitis due to bowel perforation
10	F	56	Peritonitis due to enterocolitis

**Table 2:** Analysis of immunohistochemical findings and mean grade attributed to immunohistochemical reactions for brain samples; −: negative immunoreactivity (0%); +: slight immunopositivity in spotted cells (10%); ++: immunopositivity up to one-third of the cells (33%); +++: immunopositivity up to two-thirds of the cells (70%); and ++++: strong immunopositivity in most or all cells (100%).

Ab	Case Control	Sepsis Group
Anti-iNOs	+/−	++
Anti-NOX2	+/−	++
Anti-Nitrotyrosine	−	+++
Anti-8OHdG	−	++++

## Data Availability

The data presented in this study are available on request from the corresponding author. The data are not publicly available due to privacy and could be subject to judicial investigations.

## References

[1] Singer M., Deutschman C.S., Seymour C.W., Shankar-Hari M., Annane D., Bauer M., Bellomo R., Bernard G.R., Chiche J.-D., Coopersmith C.M. (2016). The Third International Consensus Definitions for Sepsis and Septic Shock (Sepsis-3). JAMA.

[2] https://www.salute.gov.it/portale/malattieInfettive/dettaglioNotizieMalattieInfettive.jsp?lingua=italiano&menu=notizie&p=dalministero&id=6344.

[3] https://www.worldsepsisday.org/wsd2024.

[4] https://www.cdc.gov/sepsis/about/index.html.

[5] Jarczak D., Kluge S., Nierhaus A. (2021). Sepsis—Pathophysiology and Therapeutic Concepts. Front. Med..

[6] Bertozzi G., Maiese A., Passaro G., Tosoni A., Mirijello A., De Simone S., Baldari B., Cipolloni L., La Russa R. (2021). Neutropenic enterocolitis and sepsis: Towards the definition of a pathologic profile. Medicina.

[7] Berg R.M.G., Møller K., Bailey D.M. (2011). Neuro-oxidative-nitrosative stress in sepsis. J. Cereb. Blood Flow Metab..

[8] Bertozzi G., Ferrara M., Di Fazio A., Maiese A., Delogu G., Di Fazio N., Tortorella V., La Russa R., Fineschi V. (2024). Oxidative Stress in Sepsis: A Focus on Cardiac Pathology. Int. J. Mol. Sci..

[9] Vermot A., Petit-Härtlein I., Smith S.M.E., Fieschi F. (2021). NADPH Oxidases (NOX): An Overview from Discovery, Molecular Mechanisms to Physiology and Pathology. Antioxidants.

[10] Bandookwala M., Sengupta P. (2020). 3-Nitrotyrosine: A versatile oxidative stress biomarker for major neurodegenerative diseases. Int. J. Neurosci..

[11] Hemmrich K., Suschek C.V., Lerzynski G., Kolb-Bachofen V. (2003). iNOS activity is essential for endothelial stress gene expression protecting against oxidative damage. J. Appl. Physiol..

[12] Cervellati C., Pecorelli A., Saso L., Giuffrè A., Valacchi G., Maccarrone M. (2023). Chapter 4—Fluid Redox Biomarkers in Neurological Disease.

[13] Meyerholz D.K., Beck A.P. (2018). Principles and approaches for reproducible scoring of tissue stains in research. Lab. Investig..

[14] Bertozzi G., Cafarelli F.P., Ferrara M., Di Fazio N., Guglielmi G., Cipolloni L., Manetti F., La Russa R., Fineschi V. (2022). Sudden Cardiac Death and Ex-Situ Post-Mortem Cardiac Magnetic Resonance Imaging: A Morphological Study Based on Diagnostic Correlation Methodology. Diagnostics.

[15] Cecchi R., Camatti J., Bonasoni M.P., Clemente G.M., Nicolì S., Campanini N., Mozzoni P. (2024). HIF-1α expression by immunohistochemistry and mRNA-210 levels by real time polymerase chain reaction in post-mortem cardiac tissues: A pilot study. Leg. Med..

[16] Hong Y., Chen P., Gao J., Lin Y., Chen L., Shang X. (2023). Sepsis-associated encephalopathy: From pathophysiology to clinical management. Int. Immunopharmacol..

[17] Abelli J., Méndez-Valdés G., Gómez-Hevia F., Bragato M.C., Chichiarelli S., Saso L., Rodrigo R. (2022). Potential Antioxidant Multitherapy against Complications Occurring in Sepsis. Biomedicines.

[18] Di Meo S., Reed T.T., Venditti P., Victor V.M. (2016). Role of ROS and RNS Sources in Physiological and Pathological Conditions. Oxid. Med. Cell. Longev..

[19] Titheradge M.A. (1999). Nitric oxide in septic shock. Biochim. Biophys. Acta—Bioenerg..

[20] Trinh Q.D. (2022). Recent Research in Cell Stress and Microbial Infection. Microorganisms.

[21] Milhelm Z., Zanoaga O., Pop L., Iovita A., Chiroi P., Haranguş A., Cismaru C., Braicu C., Berindan—Neagoe I. (2024). Evaluation of oxidative stress biomarkers for differentiating bacterial and viral infections: A comparative study of glutathione disulfide (GSSG) and reduced glutathione (GSH). Med. Pharm. Rep. [Internet].

[22] Lorente L., Martín M.M., González-Rivero A.F., Pérez-Cejas A., Abreu-González P., Ortiz-López R., Ferreres J., Solé-Violán J., Labarta L., Díaz C. (2019). Association between DNA and RNA oxidative damage and mortality in septic patients. J. Crit. Care.

[23] Linkermann A., Green D.R. (2014). Necroptosis. N. Engl. J. Med..

[24] Lee K.H., Cha M., Lee B.H. (2021). Crosstalk between Neuron and Glial Cells in Oxidative Injury and Neuroprotection. Int. J. Mol. Sci..

[25] Fesharaki-Zadeh A. (2022). Oxidative Stress in Traumatic Brain Injury. Int. J. Mol. Sci..

[26] Ren C., Yao R.-Q., Zhang H., Feng Y.-W., Yao Y.-M. (2020). Sepsis-associated encephalopathy: A vicious cycle of immunosuppression. J. Neuroinflammation.

[27] Millán Solano M.V., Salinas Lara C., Sánchez-Garibay C., Soto-Rojas L.O., Escobedo-Ávila I., Tena-Suck M.L., Ortíz-Butrón R., Choreño-Parra J.A., Romero-López J.P., Meléndez Camargo M.E. (2023). Effect of Systemic Inflammation in the CNS: A Silent History of Neuronal Damage. Int. J. Mol. Sci..

[28] Pan S., Lv Z., Wang R., Shu H., Yuan S., Yu Y., Shang Y. (2022). Sepsis-Induced Brain Dysfunction: Pathogenesis, Diagnosis, and Treatment. Oxid. Med. Cell. Longev..

[29] Moraes C.A., Zaverucha-do-Valle C., Fleurance R., Sharshar T., Bozza F.A., d’Avila J.C. (2021). Neuroinflammation in Sepsis: Molecular Pathways of Microglia Activation. Pharmaceuticals.

[30] Gu M., Mei X.L., Zhao Y.N. (2020). Sepsis and Cerebral Dysfunction: BBB Damage, Neuroinflammation, Oxidative Stress, Apoptosis and Autophagy as Key Mediators and the Potential Therapeutic Approaches. Neurotox. Res..

[31] Bar-Or D., Carrick M.M., Mains C.W., Rael L.T., Slone D., Brody E.N. (2015). Sepsis, oxidative stress, and hypoxia: Are there clues to better treatment?. Redox Rep..

[32] Lei X.L., Zhao G.Y., Guo R., Cui N. (2022). Ferroptosis in sepsis: The mechanism, the role and the therapeutic potential. Front. Immunol..

[33] Costa I., Barbosa D.J., Benfeito S., Silva V., Chavarria D., Borges F., Remião F., Silva R. (2023). Molecular mechanisms of ferroptosis and their involvement in brain diseases. Pharmacol. Ther..

[34] Chen X., Kang R., Kroemer G., Tang D. (2021). Ferroptosis in infection, inflammation, and immunity. J. Exp. Med..

[35] Wei X.-B., Jiang W.-Q., Zeng J.-H., Huang L.-Q., Ding H.-G., Jing Y.-W., Han Y.-L., Li Y.-C., Chen S.-L. (2022). Exosome-Derived lncRNA NEAT1 Exacerbates Sepsis-Associated Encephalopathy by Promoting Ferroptosis Through Regulating miR-9-5p/TFRC and GOT1 Axis. Mol. Neurobiol..

[36] Wang J., Yang S., Jing G., Wang Q., Zeng C., Song X., Li X. (2023). Inhibition of ferroptosis protects sepsis-associated encephalopathy. Cytokine.

[37] Gou Z., Su X., Hu X., Zhou Y., Huang L., Fan Y., Li J., Lu L. (2020). Melatonin improves hypoxic-ischemic brain damage through the Akt/Nrf2/Gpx4 signaling pathway. Brain Res. Bull..

[38] Xie Z., Xu M., Xie J., Liu T., Xu X., Gao W., Li Z., Bai X., Liu X. (2022). Inhibition of Ferroptosis Attenuates Glutamate Excitotoxicity and Nuclear Autophagy in a CLP Septic Mouse Model. Shock.

[39] Corrêa S.A.L., Eales K.L. (2012). The Role of p38 MAPK and Its Substrates in Neuronal Plasticity and Neurodegenerative Disease. J. Signal Transduct..

[40] Ferrada L., Barahona M.J., Vera M., Stockwell B.R., Nualart F. (2023). Dehydroascorbic acid sensitizes cancer cells to system xc- inhibition-induced ferroptosis by promoting lipid droplet peroxidation. Cell Death Dis..

[41] Tanaka T., Mori M., Sekino M., Higashijima U., Takaki M., Yamashita Y., Kakiuchi S., Tashiro M., Morimoto K., Tasaki O. (2021). Impact of plasma 5-hydroxyindoleacetic acid, a serotonin metabolite, on clinical outcome in septic shock, and its effect on vascular permeability. Sci. Rep..

[42] Gęgotek A., Skrzydlewska E. (2022). Antioxidative and Anti-Inflammatory Activity of Ascorbic Acid. Antioxidants.

[43] Mantzarlis K., Tsolaki V., Zakynthinos E. (2017). Role of Oxidative Stress and Mitochondrial Dysfunction in Sepsis and Potential Therapies. Oxid. Med. Cell. Longev..

[44] Fowler A.A., Syed A.A., Knowlson S., Sculthorpe R., Farthing D., DeWilde C., Farthing C.A., Larus T.L., Martin E., Brophy D.F. (2014). Phase I safety trial of intravenous ascorbic acid in patients with severe sepsis. J. Transl. Med..

[45] Marik P.E., Khangoora V., Rivera R., Hooper M.H., Catravas J. (2017). Hydrocortisone, Vitamin C, and Thiamine for the Treatment of Severe Sepsis and Septic Shock: A Retrospective Before-After Study. Chest.

[46] Wu K., Liu L., Wu Z., Huang Q., Zhou L., Xie R., Wang M. (2024). Ascorbic acid induces ferroptosis via STAT3/GPX4 signaling in oropharyngeal cancer. Free Radic. Res..

[47] Szakmany T., Hauser B., Radermacher P. (2012). N-acetylcysteine for sepsis and systemic inflammatory response in adults. Cochrane Database Syst. Rev..

[48] Huang Q., Ding Y., Fang C., Wang H., Kong L. (2023). The Emerging Role of Ferroptosis in Sepsis, Opportunity or Challenge?. Infect. Drug Resist..

[49] Kumar S., Saxena J., Srivastava V.K., Kaushik S., Singh H., Abo-El-Sooud K., Abdel-Daim M.M., Jyoti A., Saluja R. (2022). The Interplay of Oxidative Stress and ROS Scavenging: Antioxidants as a Therapeutic Potential in Sepsis. Vaccines.

[50] Lambden S. (2019). Bench to bedside review: Therapeutic modulation of nitric oxide in sepsis—An update. Intensive Care Med. Exp..

[51] Kopustinskiene D.M., Bernatoniene J. (2021). Molecular Mechanisms of Melatonin-Mediated Cell Protection and Signaling in Health and Disease. Pharmaceutics.

[52] Chitimus D.M., Popescu M.R., Voiculescu S.E., Panaitescu A.M., Pavel B., Zagrean L., Zagrean A.-M. (2020). Melatonin’s Impact on Antioxidative and Anti-Inflammatory Reprogramming in Homeostasis and Disease. Biomolecules.

[53] Srinivasan V., Spence D.W., Pandi-Perumal S.R., Brown G.M., Cardinali D.P. (2011). Melatonin in mitochondrial dysfunction and related disorders. Int. J. Alzheimer’s Dis..

[54] Taha A.M., Mahmoud A.M., Ghonaim M.M., Kamran A., AlSamhori J.F., AlBarakat M.M., Shrestha A.B., Jaiswal V., Reiter R.J. (2023). Melatonin as a potential treatment for septic cardiomyopathy. Biomed. Pharmacother..

[55] Wang X. (2009). The antiapoptotic activity of melatonin in neurodegenerative diseases. CNS Neurosci. Ther..

[56] Ameri A., Asadi M.F., Ziaei A., Vatankhah M., Safa O., Kamali M., Fathalipour M., Mahmoodi M., Hassanipour S. (2023). Efficacy and safety of oral melatonin in patients with severe COVID-19: A randomized controlled trial. Inflammopharmacology.

[57] Salehi B., Mishra A.P., Nigam M., Sener B., Kilic M., Sharifi-Rad M., Fokou P.V.T., Martins N., Sharifi-Rad J. (2018). Resveratrol: A Double-Edged Sword in Health Benefits. Biomedicines.

[58] Ren Z., Wang L., Cui J., Huoc Z., Xue J., Cui H., Mao Q., Yang R. (2013). Resveratrol inhibits NF-kB signaling through suppression of p65 and IkappaB kinase activities. Pharmazie.

[59] Li J., Zeng X., Yang F., Wang L., Luo X., Liu R., Zeng F., Lu S., Huang X., Lei Y. (2022). Resveratrol: Potential Application in Sepsis. Front. Pharmacol..

[60] Lauridsen C., Jensen S.K. (2012). α-Tocopherol incorporation in mitochondria and microsomes upon supranutritional vitamin E supplementation. Genes Nutr..

[61] Dang H., Li J., Liu C., Xu F. (2021). The Association Between Vitamin E Deficiency and Critically Ill Children With Sepsis and Septic Shock. Front. Nutr..

[62] Thompson M.A., Zuniga K., Sousse L., Christy R., Gurney C.J. (2022). The Role of Vitamin E in Thermal Burn Injuries, Infection, and Sepsis: A Review. J. Burn Care Res..

[63] Bertozzi G., Maglietta F., Sessa F., Scoto E., Cipolloni L., Di Mizio G., Salerno M., Pomara C. (2020). Traumatic Brain Injury: A Forensic Approach: A Literature Review. Curr. Neuropharmacol..

[64] Wang H.-C., Lin Y.-J., Shih F.-Y., Chang H.-W., Su Y.-J., Cheng B.-C., Su C.-M., Tsai N.-W., Chang Y.-T., Kwan A.-L. (2016). The Role of Serial Oxidative Stress Levels in Acute Traumatic Brain Injury and as Predictors of Outcome. World Neurosurg..

[65] Ferrara M., Bertozzi G., Zanza C., Longhitano Y., Piccolella F., Lauritano C.E., Volonnino G., Manetti A.C., Maiese A., La Russa R. (2022). Traumatic Brain Injury and Gut Brain Axis: The Disruption of an Alliance. Rev. Recent Clin. Trials.

[66] Abdul-Muneer P.M., Chandra N., Haorah J. (2015). Interactions of oxidative stress and neurovascular inflammation in the pathogenesis of traumatic brain injury. Mol. Neurobiol..

[67] Ferrara M., Bertozzi G., Volonnino G., Di Fazio N., Frati P., Cipolloni L., La Russa R., Fineschi V. (2022). Glymphatic System a Window on TBI Pathophysiology: A Systematic Review. Int. J. Mol. Sci..

[68] Bertozzi G., Ferrara M., La Russa R., Pollice G., Gurgoglione G., Frisoni P., Alfieri L., De Simone S., Neri M., Cipolloni L. (2021). Wound Vitality in Decomposed Bodies: New Frontiers Through Immunohistochemistry. Front. Med..

